# Effect of Superfine Grinding on Antidiabetic Activity of Bitter Melon Powder

**DOI:** 10.3390/ijms131114203

**Published:** 2012-11-02

**Authors:** Ying Zhu, Ying Dong, Xiwen Qian, Fengjie Cui, Qin Guo, Xinghua Zhou, Yun Wang, Yi Zhang, Zhiyu Xiong

**Affiliations:** School of Food and Biological Engineering, Jiangsu University, Zhenjiang 212013, China; E-Mails: ying307@126.com (Y.Z.); xwqian@126.com (X.Q.); fengjiecui@163.com (F.C.); guoqin_shiyin@163.com (Q.G.); xinghuazhou638@163.com (X.Z.); wangy1974@ujs.edu.cn (Y.W.); zhangyihahahei@126.com (Y.Z.); xzy19880304@163.com (Z.X.)

**Keywords:** bitter melon powder, diabetes, superfine grinding, anti-diabetic activity, food processing

## Abstract

The antidiabetic activities of bitter melon powders produced with lyophilization/superfine grinding and hot air drying/normal grinding were investigated *in vivo* for selecting a suitable bitter melon processing procedure. After a five-week treatment, bitter melon lyophilized superfine grinding powder (BLSP) had a higher antidiabetic activity with reducing fasting blood glucose levels from 21.40 to 12.54 mmol/L, the serum insulin levels from 40.93 to 30.74 mIU/L, and restoring activities of SOD compared with those in the bitter melon hot air drying powder (BAP) treated group. Furthermore, BLSP protected pancreatic tissues including islet beta cells and reduced the loss of islet cells. Combined with the difference of compositions in BLSP and BAP, it could be concluded that superfine grinding and lyophilization processes were beneficial for presenting the antidiabetic activity, which will provide a reference for direct utilization of bitter melon as a suitable functional food to relieve symptoms of diabetes.

## 1. Introduction

*Momordica charantia* Linn. (Cucurbitaceae) is referred to as bitter melon or bitter gourd and has recently attracted considerable attention for its various physiological activities, such as its antitumor [1–3], anti-inflammatory [4], antioxidant [5,6], antibacterial [7,8], hypoglycemic [9–11], hypocholesterolemic [12], hypotriglyceridemic [13], and immunostimulating activities [14]. Previous investigations have shown that the fruits and leaves of *M. charantia* had rich phenolics and exhibited a high antioxidant activity [5]. Nowadays, it has been used as a traditional antidiabetic remedy in eastern countries and areas for many years [11,15]. Fresh bitter melon is also used as a nourishing food, as it contains: 93.8% water, 0.9% protein, 0.1% lipid, 3.3% dietary fiber, 20 kJ energy per 100 g, 0.6% ash, and a small quantity, 0.05%, of vitamin C [16].

The antidiabetic evaluation of bitter melon has been well investigated in streptozocin- or alloxan-induced diabetic rats, mice and rabbits, high-fat diet-induced obesity mice, as well as in humans with type 2 diabetes [9,13,17–20]. The hypoglycemic potential components in bitter melon have been identified as glycosides, saponins, alkaloids, triterpenes, polysaccharides, proteins, and steroids [14,21]. Although several pure chemicals were isolated from bitter melon and applied for investigating their antidiabetic mechanisms, the mixture of these hypoglycemic chemicals such as saponins or charantins seemed to present a significantly higher bioactivity. For example, the hypoglycemic chemicals of bitter melon are proven as a mixture of steroidal saponins known as charantins and alkaloids [21]. The antidiabetic mechanisms of bitter melon also have been proposed. Bitter melon has shown to stimulate glycogen storage by liver and insulin secretion by islets of Langerhans [13,22]. Bitter melon suppresses weight gain and has the potential to reduce adiposity [23]. Moreover, bitter melon supplementation lowered serum and hepatic triglyceride in normal rats [24]. Bitter melon may possess insulin-like properties, preserved pancreatic islet beta cells [23,25]. A recent study proved that bitter melon could upregulate the significance of glucose transporter 4 (GLUT-4), peroxisome proliferator-activated receptor γ (PPARγ) and phosphatidylinositol 3 kinase (PI3K) by augmenting the glucose uptake and homeostasis [26]. Bitter melon can also improve insulin sensitivity by increasing insulin-stimulated insulin receptor substrate-1 (IRS1) tyrosine phosphorylation in high-fat diet-fed mice/rats [27,28]. Hence, the synergic effect of these bioactive components would possibly make the contribution. Therefore, the question is: “Do we need to evaluate the antidiabetic activity of bitter melon by using purified samples?”

Superfine grinding technology is a new technology and a useful tool for preparing superfine powder [29]. Compared with other samples ground with traditional mechanical methods, superfine powder bears good physical properties like dispersibility and solubility. To date, the superfine grinding technology has also been applied in biotechnology and foodstuffs and shown a high potential for many other commercial applications [30]. For example, the *Astragalus membranaceus* powder obtained with superfine grinding had high water-holding capacity, high fluidity, high water solubility index and high protein solubility [31]. Now, Chinese markets sell the bitter melon powder processed by hot drying and milling which might result in the inactivation of components in the samples. Nevertheless, lyophilized bitter melon had also previously been superfine-ground with its particle size less than 50 μm by our group. Similar to other foodstuffs, superfine grinding bitter melon powder after the lyophilization process retains the whole chemical compositions of fresh samples, such as proteins, polysaccharides, glycosides, saponins, alkaloids, and triterpenes, while its physical properties received significant changes after the grinding process. However, few references are presented for evaluating the antidiabetic activity of superfine grinding bitter melon powder containing a total of bioactive compositions.

The objectives of this study were: (1) to obtain the bitter melon lyophilized superfine grinding powder (BLSP) and bitter melon hot air drying superfine grinding powder (BAP); (2) to compare their differences in physical/chemical properties, antidiabetic activity and their mechanisms *in vivo*; and, (3) to conclude the processing effect on the bioactivity of bitter melon.

## 2. Results and Discussion

### 2.1. Physical and Chemical Properties of Superfine-Ground Bitter Melon

**Figure 1:**
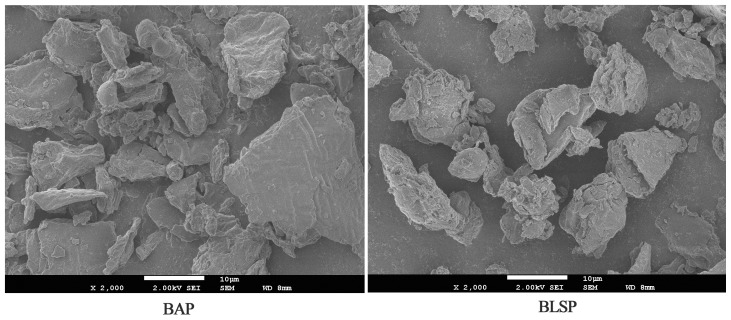
SEM images of BAP and BLSP.

Figure 1 presented the morphology of fragmented bitter melon powder. BLSP had the better uniformity with a particle size of 3–10 μm, while the particle size of BAP was 50–150 μm. After superfine grinding, the water content of the powder reduced from 5.92% to 2.66%, and the water solubility index increased from 37.82% to 39.38% (Table 1).

The chemical composition of BLSP and BAP are shown in Table 2. BLSP had higher total polyphenols of 10.03 mg/g and total flavonoids of 5.27 mg/g compared with BAP (*p* < 0.05). Meanwhile, the decrease in particle size of bitter melon resulted in the increase of the water-soluble sugar levels and the water-soluble protein contents (*p* < 0.05). The total saponins content of BLSP was 2.74%, significant higher than BAP (*p* < 0.05). These results imply that superfine grinding is helpful for improving the extraction efficiency of the nutritional components of bitter melon.

Temperature can promote oxidation of polyphenols and flavanoids, BAP was processed by 60 °C hot air drying while BLSP was lyophilized (the high temperature was 5 °C). Hence, the contents of polyphenols and flavanoids in BAP are lower than BLSP. The powder with particle size less than 10–25 μm was defined as superfine material and possessed a narrow and uniform particle size distribution with good surface area that ensure easy dispersibility, solubility and flowability [29,32]. Nowadays, the superfine grinding technology has been widely used in the food processing industry. The superfine grinding could cause marked differences in chemical composition separation of the granulometric fractions [33], and the hydration rate and the bioavailability of materials increased [34]. This helps to promote the digestion and absorption of samples. For example, superfine grinding affects the composition and hydration properties of the wheat bran dietary fiber (DF) products with the decrease of particle size [35]. Superfine green tea had a better water content of 4.52%, held the stable green and bright color, and increased the concentrations of other components including catechins, tea polysaccharide [36]. In this study, the contents of total polyphenols, total flavonoids, total saponins, water-soluble protein and sugar in BLSP were higher than those in BAP. The results suggest that superfine grinding is helpful for releasing the nutritional components from bitter melon. However, we have not given the detailed chemical analysis of these major ingredients in this paper, such as polyphenols, flavonoids and saponins; in future experiments, we will focus on these by HPLC/HPLC-MS.

Superfine grinding also improves the bioactivities of experimental materials. For example, the superfine wheat bran DF had increased antioxidant activities including chelating activity, reducing power and total phenolic content (TPC) [35]. A similar conclusion was also obtained that superfine grinding markedly increased extraction of tea polysaccharide (TPS) and that of the potent scavenging capacity of green tea powder (GTP) [36]. The present study presented the antidiabetic differences between bitter melon powders after lyophilization/superfine grinding and hot air drying/normal grinding processes for the first time.

### 2.2. Effect of Processing Methods of Bitter Melon on Body Weight, Food and Water Intakes in Control and Diabetic Rats

**Figure 2:**
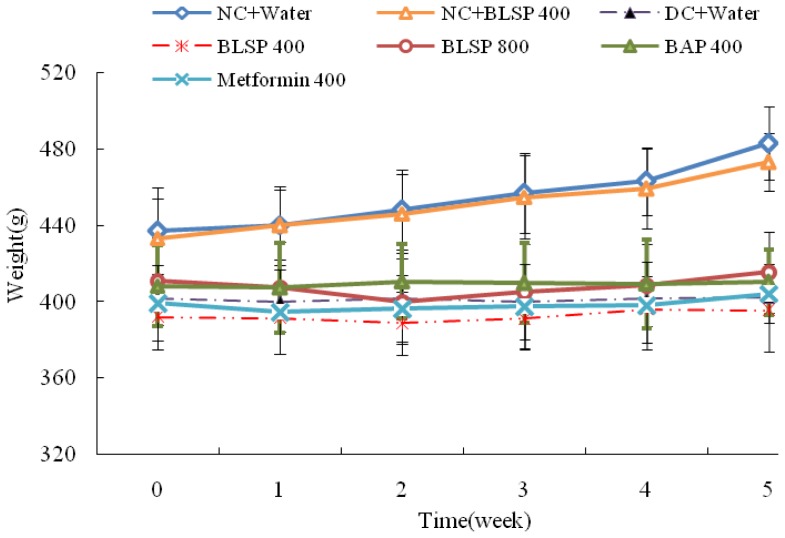
Effect of BLSP and BAP on mean weekly body weight in diabetic rats. Values are expressed as means ± SD (*n* = 6). Statistical analysis was performed using ANOVA. The means with different superscript were considered significantly different (*p* < 0.05).

The changes in body weight are shown in Figure 2. At the beginning of the experiment, body weights of the diabetic groups were lower than those of two control groups, but with no statistical differences (*p* > 0.05). After a five-week treatment, bitter melon did not affect the body weight of diabetic groups significantly while body weights of the two normal groups increased significantly from 430 g to 480 g (*p* < 0.05) and the nontreated diabetic group only increased the body weight by 0.82 g (Table 3). BLSP and BAP treatment had no effect on daily water intake. The diabetic rats treated with 800 mg/kg/day BLSP significantly reduced their food intake and improved on food efficiency ratio (food efficiency ratio = body weight gain/food intake × 100%) as compared with the DC group rats (*p* < 0.05) [37–39]. Several studies have reported that feeding rats a high-fat diet leads to the development of insulin resistance [40,41]. STZ can selectively target and destroy the pancreatic islet beta cells and also can make cells less active [42]. The effects of STZ on glucose and insulin homeostasis reflect the toxin-induced abnormalities in beta cell function. Srinivasan *et al.* developed the diabetic rat model by the combination of high-fat diet-fed and low-dose STZ-treated; this model replicated the natural history and metabolism of human Type 2 diabetes [43]. The experimental diabetic rat model used in this study was induced by a high-fat diet and low-dose STZ of 35 mg/kg bw, which closely mimic the metabolic characteristics of the type 2 diabetes in humans [37–39]. Results from this study show that the diabetic rats’ body weights were significantly decreased (*p* < 0.5) compared with those of NC group. The weight loss might be associated with the abnormalities in carbohydrate, fat, and protein metabolism, which was confirmed from the water intake and food intake data.

Kasbia *et al.* found that a single dose of freeze-dried bitter melon 50 mg/kg and 100 mg/kg given to healthy volunteers did not reduce fasting glucose or plasma glucose after OGTT [44]. Therefore, the dosage of 400 mg/kg bw was selected in the present study due to its equivalence to an adult (60 kg) taking 24 g BLSP (about 384 g fresh bitter melon) per day, which is acceptable for the adult daily intake for the antidiabetic effect, but with no adverse effects.

### 2.3. Effect of Processing Methods of Bitter Melon on Fasting Blood Glucose Levels (FBG) and Insulin Levels

Table 4 presented the effect of BLSP and BAP on fasting blood glucose (FBG) levels in various experimental groups. At the beginning of the study, the FBG levels of all diabetic groups were approximately 20 mmol/L, which were fourfold over the NC group. At the end of fifth week, the FBG levels of normal rats treated with distilled water kept a stable level of 5.00 mmol/L while those of normal rats treated with 400 mg/kg/day of BLSP were reduced to 4.40 mmol/L (*p* > 0.05). All of BLSP-, BAP- and metformin-treated diabetic groups had a reduction of the FBG levels when compared with DC group. The treatment with 800 mg/kg/day of BLSP or 400 mg/kg/day of metformin could significantly decrease the FBG levels (*p* < 0.05). Of the four diabetic-treatment groups, the 800 mg/kg/day of BLSP was found to be the most effective in reducing FBG levels.

Table 4 also gave the changes of insulin levels between experimental groups treated with or without BLSP, BAP and metformin. High-fat diet feeding combined with STZ injection caused abnormal insulin secretion which finally increased the serum insulin levels of the diabetic groups significantly compared with NC group (*p* < 0.05). The administration of BLSP and metformin with the dosage of 400 mg/kg/day significantly reduced the serum insulin levels compared with DC group (*p* < 0.05). However, BAP at the dosage of 400 mg/kg/day had no similar activity for balancing the serum insulin levels.

High-fat diet and STZ resulted in increases in blood glucose levels and insulin levels in the DC group. After the rats were administered BLSP, BAP and metformin for five weeks, the FBG levels of the BLSP and metformin groups improved significantly, while the BAP group decreased slightly. The increased levels of serum insulin in diabetic rats were significantly lowered by the administration of BLSP and metformin, especially the low dose group of BLSP, which were in agreement with the results obtained by Jayasooriya *et al.* with a marked reduction of serum glucose concentration in the freeze-dried bitter melon powder-treated group [12]. The reduction of glucose levels and serum insulin levels suggest that BLSP might have a better effect in increasing the renewal of beta cells in the pancreas, allowing for the recovery of partially destroyed beta cells [25], or displaying insulin-like properties than BAP. Metformin improves hyperglycemia primarily by suppressing glucose production by the liver, or increases insulin sensitivity, enhances peripheral glucose uptake (for example, by phosphorylating GLUT-4 enhancer factor), increases fatty acid oxidation, and decreases absorption of glucose from the gastrointestinal tract [45,46]. The antihyperglycemic differences between BLSP and BAP may be due to the different content of bioactive compounds, such as polypeptides, polyphenolics, terpenoids, which are consistent with the conclusions from Kunyanga *et al.* that high phenolic content in the foodstuffs resulted in relatively higher antioxidant and antidiabetic activities [47], and those from Keller *et al.* that saponins isolated from *M. charantia* can stimulate insulin secretion *in vitro*[48].

### 2.4. Effect of Processing Methods of Bitter Melon on Serum Lipids in Diabetic Rats

Diabetic rats had a significant higher serum TG level when compared to normal rats (*p* < 0.05). After being fed with 400 mg/kg/day of BLSP, the TG concentrations in diabetic rats were reduced to a significant lower level (0.67 mmol/L) than those in the DC group rats (1.03 mmol/L) (*p* < 0.05) (Table 5). Moreover, CHOL and LDL levels increased with a decrease in the HDL levels in the diabetic groups, although no statistical differences were observed (*p* > 0.05). From the results, it is clear that the administration of BLSP had a better effect on regulating serum lipid than BAP and metformin.

Type 2 diabetes mellitus is associated with profound alterations in the plasma lipid and lipoprotein profile. The levels of TG, LDL and CHOL increase while the HDL levels decline [49]. Bitter melon reduces serum TG level. Furthermore, the obtained data proved that BLSP with 400 mg/kg/day could reduce the serum TG level significantly, which is consistent with those obtained by Senanayake *et al.*, by using methanol to extract the active components from bitter melon [24]. However, serum cholesterol levels in various experimental groups had a slight difference from those of Ahmed *et al.*[13]. The changes in bioactive components concentrations released from bitter melons might make the contribution. In the present study, BLSP can better ameliorate serum TG level of rats than BAP, which also proved that powder size and lyophilization can improve the release of active components, as well as retaining their bioactivity [50]. The main components to make this contribution may be saponins. Popovich *et al.* found that bitter melon triterpenoid extract reduces lipid accumulation and adiponectin expression in 3T3-L1 cells [51].

### 2.5. Effect of Processing Methods of Bitter Melon on MDA Levels and the SOD and GPx Activities in Diabetic Rats

The MDA levels and the activities of enzymatic antioxidants (SOD and GPx) in the livers were determined (Table 6). The treatment of BLSP, BAP and metformin had no obvious effect on reducing MDA levels and improving the activities of GPx of the diabetic groups. However, the activities of SOD in the BLSP 800 group and Metformin 400 group significantly increased as compared to the DC group (*p* < 0.05), which was close to the levels of the NC group.

### 2.6. Histologic Findings

**Figure 3:**
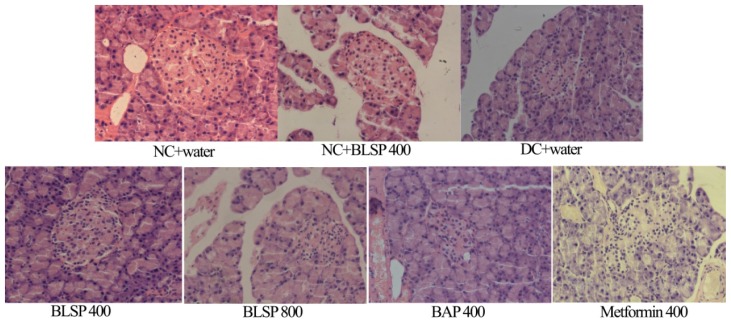
Histopathological analysis of Langerhans islets of diabetic rat pancreas (original magnification 400×). The pancreatic sections were stained with hematoxylin and eosin stain (H and E).

**Figure 4:**
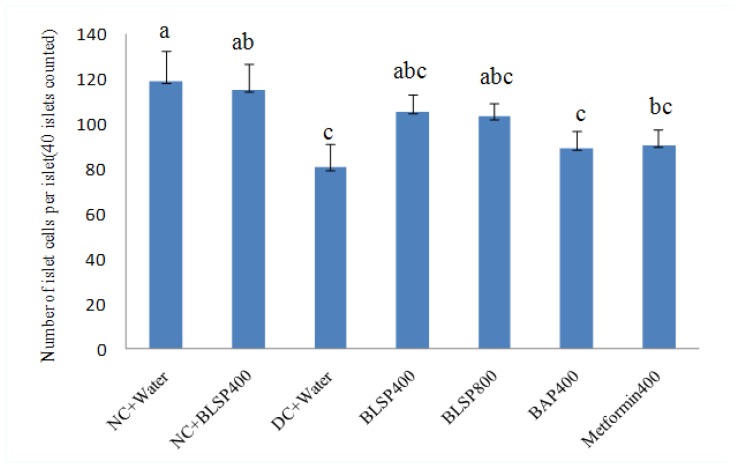
Effect of BLSP and BAP on islet cell mass in diabetic rats. Values are expressed as means ± SD (*n* = 6). Statistical analysis was performed using ANOVA. The means with different superscript were considered significantly different (*p* < 0.05).

Figure 3 presented the histologic findings of the pancreas in the diabetic rats. From Figure 3, the pancreas was partially damaged by STZ, including atrophy and multiple irregular projections of islets. When diabetic animals were treated with BLSP and BAP for five weeks, the islets were partially renovated, especially in the BLSP 400 group. There is no difference of the relative islet cell mass in the rat pancreas among the BLSP, BAP and metformin groups (*p* > 0.05), while the increase of islet cell mass is significant compared with the untreated diabetic rats (Figure 4).

The interesting observation in this research was the pathophysiologic changes of islets in diabetic rats treated with or without bitter melon samples. Diabetic rats had the islets with a relatively decreased population of insulin-producing beta cells and multiple irregular projections [52]. In the BLSP 400 group, pancreatic islets showed predominantly insulin-producing beta cells with basophilic cytoplasm and a few eosinophilic glucagon-producing alpha cells, and also the arrangement of the endocrine cells in the islets appeared similar to normal. Ahmed *et al.* also found that bitter melon fruit juice may have a role in the renewal of beta cells in STZ-diabetic rats [25]. However, BAP 400 group and Metformin 400 group did not show any significant effect on renovating islets of the diabetic rats. Pinent *et al.* have correlated flavonoid intake to increases in beta cell mass, with the flavonoids either inhibiting apoptosis or promoting proliferation of beta cells [53]. Bitter melon is a rich source of flavonoids, and this may contribute to the BLSP capacity to renovate the islets of rats. Furthermore, the difference between BLSP and BAP can be due to the difference in the changes of nutritious components (such as flavonoids) released based on the different particle sizes.

It was evident in the study that high-fat diet and STZ-induced rats had hyperlipidaemia and hypertriglyceridemia [38,54], while BLSP-treated rats had reduced glucose concentrations and improved insulin sensitivity. The main mechanism may be that bitter melon can regulate PPARα-mediated pathway or influence PPARγ-mediated pathway [40]. In addition, the main nutrition components associated with the effects may be pholenols, flavonoids and saponins. We speculate that BLSP could be another choice in the protection against visceral obesity and type 2 diabetes.

## 3. Experimental Section

### 3.1. Plant Material

The fresh fruits of wild bitter melon were obtained from Lvjian Agricultural Station (Yangzhong City, China) and were authenticated by Jiangsu Academy of Agricultural Science. The bitter melon selected for the present study had a 20–25 mm diameter and green appearance.

Bitter melon lyophilized superfine grinding powder (BLSP) was prepared by washing the unripe bitter melons with tap water, removing the seeds, lyophilizing the remaining portion with the pressure of 30 Pa for 24 h at 5 °C, and superfinely ground with a HSF high-speed hammer mill (National Special Superfine Powder Engineering Research Center of China, Nanjing, China). The preparation methods of bitter melon hot air drying superfine grinding powder (BAP) was similar to those of the BLSP, except the 60 °C hot air procedure (24 h) and grounding with a QE-100g mill (Zhejiang Yili Co., Jinghua, China). BAP and BLSP were filtered through a 150 μm screen before further experiments. The obtained samples were stored at 4 °C for further use.

### 3.2. Determination of Physical Properties of BLSP and BAP

The Morphological characterization of BLSP and BAP was analyzed with a Field Emission Scanning Electron Microscope (FE-SEM: Jeol JSM-7001F: with Au-coated, operated at 10 kW). Particle size distribution of BLSP and BAP were measured by a laser diffraction instrument (Mastersizer 2000, UK). The moisture contents were determined by using a HB43-S moisture analyzer (Mettler Toledo, Switzerland). The water activity (Aw) values were determined by using AQUA Lab (Decagon Devices, Inc., USA). The water solubility index of 20 min was determined by using the method described by Zhao *et al.*[55].

### 3.3. Analysis of Chemical Components of BLSP and BAP

Two grams of powders were extracted with 100 mL distilled water at 100 °C for 30 min, filtering the infusions to obtain the resulting supernatant. After cooling and filtering, the volume was made up to 100 mL with distilled water. Water-soluble nitrogen content was analyzed with the Bradford procedure based on an established calibration curve. The results were expressed as BSA (Bovine serum albumin) equivalents in mg/g dry material. Additionally, water-soluble sugar content was analyzed by using the anthranone reagent and the results were expressed as d-glucose equivalents percent. The total of polyphenols was analyzed by the Folin-Ciocalteu method. Briefly, this solution (0.5 mL) was mixed with 2.5 mL of distilled water, 1.5 mL of 7.5% sodium carbonate (Na_2_CO_3_), and 0.5 mL of Folin-Ciocalteau reagent. After incubation at 45 °C for 30 min, the absorbance of the reaction mixture absorbance was measured at 750 nm, and the content was expressed as gallic acid equivalents in mg/g dry material.

The total flavonoid content was determined according to the aluminum chloride colorimetric method [6]. The total flavonoid content was expressed in milligrams of rutin equivalents per gram of dry material. The total saponin content was determined used the method described by Xu & Dong [56] and expressed as Ginsenoside Rg1 equivalents percent.

### 3.4. Animals

Sprague–Dawley male rats weighing 200 ± 20 g were obtained from the Laboratory Animal Research Center of Jiangsu University (LARC, Zhenjiang, China) with the license number SCXK (SU) 2009–0002 and SYXK (SU) 2008–0024. The rats were caged individually in LARC at temperature of 22 ± 2 °C and a relative humidity of 40%–60%, and artificially illuminated on an approximate 12 hrlight/dark cycle. The air exchange was about 18 times/h. All the rats were provided food and filtered tap water *ad libitum*.

### 3.5. Induction of Diabetes

Rats were randomly separated into two groups: normal control (NC) and diabetic groups. The NC group was fed with a basic diet and experimental animals were fed with a high-fat and high-sucrose diet (containing of 7% lard, 15% sucrose and 78% basic diet) for 4 weeks. After feeding the rats with these diets for 4 weeks, the rats were then made to fast overnight before treatment. Type 2 diabetic rats were induced by a single intraperitoneal injection of STZ (Sigma Chemical Co., St. Louis, MO, USA) freshly dissolved in a 0.1 mol/L citrate buffer (pH 4.5) at a dosage of 35 mg/kg body weight. The NC group was administered with citrate buffer (pH 4.5). Three days later, the rats were confirmed as the diabetic model when their fasting plasma glucose levels exceeded 11.1 mmol/L. Diabetes were stabilized in these STZ-treated rats over a period of 7 days before the experiment.

### 3.6. Experimental Design

Experimental rats with 12 of normal model and 30 of STZ-diabetic model were divided into 7 groups of 6 each. Group 1 (NC + water) consisted of normal rats treated with distilled water (4 mL/kg body weight); Group 2 (NC + BLSP 400) consisted of normal rats treated with BLSP (400 mg/kg bw); Group 3 (DC (diabetic control) + water) consisted of diabetic rats treated with distilled water (4 mL/kg bw); Group 4 (BLSP 400) consisted of diabetic rats treated with BLSP (400 mg/kg bw); Group 5 (BLSP 800) consisted of diabetic rats treated with BLSP (800 mg/kg bw); Group 6 (BAP 400) consisted of diabetic rats treated with BAP (400 mg/kg bw); Group 7 (Metformin 400) consisted of diabetic rats treated with 400 mg/kg bw of metformin (Beijing Jingfeng Pharmaceutical CO., LTD, Beijing, China).

During the five-week treatment, the body weight of each rat and the food/water intake volumes were measured weekly, then the animals were anesthetized with chloral hydrate. Blood samples were collected via abdominal aorta puncture. The blood samples were centrifuged at 3500 rpm for 10 min to obtain the serum, which was kept at −20 °C for further analysis.

### 3.7. Blood Biochemical Assays

Fasting blood glucose was measured using OneTouch Ultra Blood Glucose Meter (Johnson & Johnson Medical (China) Ltd., Shanghai, China).

The sera were assayed for triglycerides (TG), cholesterol (CHOL), high-density lipoproteins cholesterol (HDLC) and low-density lipoproteins cholesterol (LDLC) levels with an Olympus AU2700 Clinical Chemistry Analyzer (Olympus Inc., Japan). The insulin levels were determined using commercial rat insulin ELISA kits (R & D Systems China Co. Ltd., Shanghai, China).

### 3.8. Oxidative Stress Markers and Antioxidant Enzymes

The end product of liver tissue lipid peroxidation quantity was expressed by the content of the malondialdehyde (MDA). The MDA content was determined using commercially available kits (Nanjing Jiancheng Bio CO., Nanjing, China). While the levels of SOD and glutathione peroxidase (GPx) of the liver tissue were also determined using commercially available kits (Nanjing Jiancheng Bio CO., Nanjing, China).

### 3.9. Histopathologic Procedures

Pancreatic tissues were harvested from the sacrificed rats. The tissues were fixed in 10% neutral buffered formalin, embedded in paraffin, cut to approximately 4 μm sections, and stained with hematoxylin and eosin (H&E). The slides were viewed on a Zeiss Axiovert 40 Microscope (Carl Zeiss, Oberkochen, Germany).

### 3.10. Statistical Analyses

The data were presented as group mean values ± SD (standard deviation) and were analyzed by one-way analysis of variance (ANOVA). All the statistical analyses were performed using SPSS v14.0 (SPSS Inc., Chicago, IL, USA). *p* values <0.05 were considered as significant.

## 4. Conclusions

Superfine grinding and lyophilization are helpful for improving extraction of proteins, polysaccharides and other bioactive components such as total pholenols, flavonoids and saponins from bitter melon, which might attribute to the more significant blood glucose- and TG- lowering effect and the renovated islets of rats with high-fat diet and STZ- induced diabetes than those of BAP. These findings also provided a direct utilization method of bitter melon as a suitable functional food to relieve symptoms of diabetes. Further investigations will be focused on investigating the relationship of the antihyperglycemic molecular mechanism of BLSP with particle size and proposing an energy-saving grinding process.

## Figures and Tables

**Table 1:** Physical analysis of BLSP and BAP.

Sample	Mean diameter (μm)	Moisture (%)	Aw	Water solubility index (%)
BLSP	6.5	2.66 ± 0.05 ^b^	0.37 ± 0.01 ^a^	39.38 ± 0.04 ^a^
BAP	100	5.92 ± 0.06 ^a^	0.34 ± 0.01 ^a^	37.82 ± 0.03 ^b^

Values are expressed as means ± SD (*n* = 3). Statistical analysis was performed using ANOVA. The means with different superscript were considered significantly different (*p <* 0.05).

**Table 2:** Contents of main chemical components of BLSP and BAP.

Sample	Water-soluble protein (mg/g)	Water-soluble sugar (%)	Total polyphenols (mg/g)	Total flavonoids (mg/g)	Total saponins (%)
BLSP	2.99 ± 0.04 ^a^	13.07 ± 0.11 ^a^	10.03 ± 0.02 ^a^	5.27 ± 0.05 ^a^	2.74 ± 0.01 ^a^
BAP	2.17 ± 0.06 ^b^	11.01 ± 0.09 ^b^	6.43 ± 0.04 ^b^	2.09 ± 0.04 ^b^	2.00 ± 0.02 ^b^

Values are expressed as means ± SD (*n* = 3). Statistical analysis was performed using ANOVA. The means with different superscript were considered significantly different (*p* < 0.05).

**Table 3:** Effect of BLSP and BAP on body weight gain, food intake, water intake in diabetic rats.

Group	BW gain (g)	Food intake (g/d)	Food efficiency ratio (%) *	Water intake (mL/d)
NC + Water	45.82 ± 6.35 ^a^	23.74 ± 1.98 ^c^	5.49 ± 0.11 ^a^	45.89 ± 5.55 ^b^
NC + BLSP 400	39.81 ± 4.87 ^a^	23.31 ± 0.34 ^c^	5.28 ± 0.05 ^b^	38.11 ± 2.58 ^b^
DC + Water	0.82 ± 0.40 ^c^	48.20 ± 1.46 ^a^	0.05 ± 0.01 ^e^	174.75 ± 23.52 ^a^
BLSP 400	3.60 ± 0.80 ^b^	42.43 ± 2.58 ^ab^	0.24 ± 0.01 ^d^	161.05 ± 34.87 ^a^
BLSP 800	4.80 ± 0.64 ^b^	40.86 ± 4.11 ^b^	0.34 ± 0.01 ^cd^	155.10 ± 39.56 ^a^
BAP 400	2.07 ± 0.66 ^c^	45.16 ± 4.58 ^ab^	0.14 ± 0.01 ^e^	169.42 ± 33.33 ^a^
Metformin 400	5.05 ± 0.71 ^b^	40.96 ± 3.23 ^b^	0.35 ± 0.01 ^c^	161.91 ± 27.14 ^a^

Values are expressed as means ± SD (*n* = 6). Statistical analysis was performed using ANOVA. The means with different superscript were considered significantly different (*p* < 0.05).

* Food efficiency ratio was calculated as body weight gain/food intake × 100%.

**Table 4:** Effect of BLSP and BAP on fasting blood glucose (FBG) changes and insulin levels in diabetic rats.

Group	FBG Week 0 (mmol/L)	FBG Week 5 (mmol/L)	Insulin Week 5 (mIU/L)
NC + Water	4.90 ± 0.38 ^b^	5.00 ± 0.60 ^d^	32.49 ± 1.48 ^cd^
NC + BLSP 400	4.68 ± 0.54 ^b^	4.40 ± 0.26 ^d^	33.47 ± 1.35 ^cd^
DC + Water	22.00 ± 2.91 ^a^	23.98 ± 1.85 ^a^	40.93 ± 1.31 ^a^
BLSP 400	19.25 ± 3.01 ^a^	14.85 ± 0.82 ^bc^	30.74 ± 0.38 ^d^
BLSP 800	21.40 ± 2.31 ^a^	12.54 ± 2.55 ^c^ (**)	35.45 ± 2.56 ^bc^
BAP 400	18.65 ± 3.08 ^a^	17.17 ± 1.40 ^b^	37.88 ± 2.24 ^ab^
Metformin 400	18.62 ± 3.49 ^a^	12.12 ± 1.96 ^c^ (*)	33.78 ± 0.99 ^bcd^

Values are expressed as means ± SD (*n* = 6). Statistical analysis was performed using ANOVA. The means with different superscript were considered significantly different (*p* < 0.05).

* significantly different from week 0 (*p* < 0.05).

** significantly different from week 0 (*p* < 0.01).

**Table 5:** Effect of BLSP and BAP on serum lipid profiles in diabetic rats (week 5).

Group	TG (mmol/L)	CHOL (mmol/L)	HDL (mmol/L)	LDL (mmol/L)
NC + Water	0.53 ± 0.17 ^b^	1.44 ± 0.19 ^a^	1.35 ± 0.23 ^a^	0.38 ± 0.04 ^a^
NC + BLSP 400	0.50 ± 0.07 ^b^	1.35 ± 0.22 ^a^	1.26 ± 0.20 ^a^	0.36 ± 0.09 ^a^
DC + Water	1.03 ± 0.27 ^a^	1.62 ± 0.11 ^a^	1.07 ± 0.08 ^a^	0.42 ± 0.05 ^a^
BLSP 400	0.67 ± 0.07 ^b^	1.52 ± 0.31 ^a^	1.18 ± 0.33 ^a^	0.39 ± 0.12 ^a^
BLSP 800	0.74 ± 0.14 ^ab^	1.53 ± 0.34 ^a^	1.28 ± 0.08 ^a^	0.38 ± 0.08 ^a^
BAP 400	0.85 ± 0.02 ^ab^	1.60 ± 0.27 ^a^	1.14 ± 0.19 ^a^	0.39 ± 0.08 ^a^
Metformin 400	0.88 ± 0.30 ^ab^	1.56 ± 0.36 ^a^	1.13 ± 0.24 ^a^	0.41 ± 0.10 ^a^

Values are expressed as means ± SD (*n* = 6). Statistical analysis was performed using ANOVA. The means with different superscript were considered significantly different (*p* < 0.05).

**Table 6:** Effect of BLSP and BAP on liver MDA levels, the activities of SOD and GPx in diabetic rats (week 5).

Group	MDA (nmol/mgprot)	SOD (U/mgprot)	GPx (μmol/min/L)
NC + Water	27.71 ± 1.50 ^a^	407.57 ± 55.15 ^ab^	269.83 ± 37.76 ^a^
NC + BLSP 400	27.44 ± 1.65 ^a^	453.63 ± 57.65 ^a^	227.66 ± 14.37 ^a^
DC + Water	31.19 ± 3.97 ^a^	291.73 ± 39.16 ^c^	262.47 ± 36.93 ^a^
BLSP 400	29.47 ± 1.94 ^a^	341.22 ± 58.21 ^bc^	245.25 ± 35.41 ^a^
BLSP 800	30.71 ± 5.24 ^a^	397.04 ± 23.48 ^ab^	236.01 ± 65.47 ^a^
BAP 400	31.67 ± 7.01 ^a^	304.51 ± 65.65 ^c^	278.42 ± 48.75 ^a^
Metformin 400	30.55 ± 2.95 ^a^	416.76 ± 45.47 ^ab^	253.30 ± 22.25 ^a^

Values are expressed as means ± SD (*n* = 6). Statistical analysis was performed using ANOVA. The means with different superscript were considered significantly different (*p* < 0.05).
